# Optimized Polymeric Membranes for Water Treatment: Fabrication, Morphology, and Performance

**DOI:** 10.3390/polym16020271

**Published:** 2024-01-18

**Authors:** Avneesh Kumar, Dong Wook Chang

**Affiliations:** Department of Industrial Chemistry, ECS Core Research Institute, Pukyong National University, Busan 48513, Republic of Korea; avneesh@pknu.ac.kr

**Keywords:** supramolecular assemblies, functional polymers, membranes, water purification, pollutants in water

## Abstract

Conventional polymers, endowed with specific functionalities, are extensively utilized for filtering and extracting a diverse set of chemicals, notably metals, from solutions. The main structure of a polymer is an integral part for designing an efficient separating system. However, its chemical functionality further contributes to the selectivity, fabrication process, and resulting product morphology. One example would be a membrane that can be employed to selectively remove a targeted metal ion or chemical from a solution, leaving behind the useful components of the solution. Such membranes or products are highly sought after for purifying polluted water contaminated with toxic and heavy metals. An efficient water-purifying membrane must fulfill several requirements, including a specific morphology attained by the material with a specific chemical functionality and facile fabrication for integration into a purifying module Therefore, the selection of an appropriate polymer and its functionalization become crucial and determining steps. This review highlights the attempts made in functionalizing various polymers (including natural ones) or copolymers with chemical groups decisive for membranes to act as water purifiers. Among these recently developed membrane systems, some of the materials incorporating other macromolecules, e.g., MOFs, COFs, and graphene, have displayed their competence for water treatment. Furthermore, it also summarizes the self-assembly and resulting morphology of the membrane materials as critical for driving the purification mechanism. This comprehensive overview aims to provide readers with a concise and conclusive understanding of these materials for water purification, as well as elucidating further perspectives and challenges.

## 1. Introduction

**Figure 1:**
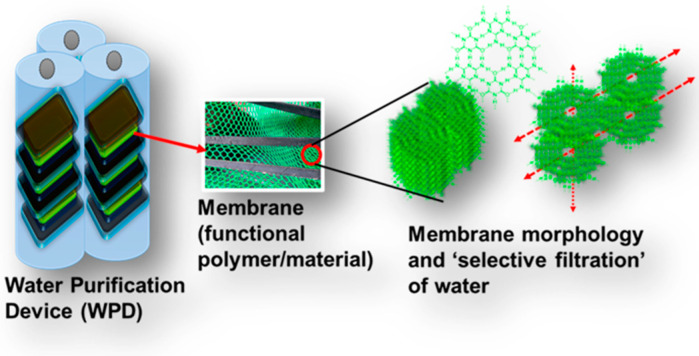
A membrane purifier comprising a functional polymer or material fabricated as a membrane and integrated within a device. The microscopic structure demonstrates the pivotal role of “specific” chemical functionality in governing the water filtration process, selectivity, and flux.

Human activities and interventions in ecosystems have led to numerous global challenges [1,2]. Among these, the availability of freshwater for drinking stands out as a significant concern. Scarcity of potable water in many regions worldwide presents a formidable challenge for sustaining life on this planet [3]. Large quantities of chemicals from industrial plants and households are dumped into rivers and other natural water reservoirs [4]. These chemicals or pollutants mainly comprise heavy metal ions, dyes, and other organic contaminants. They accumulate in water bodies, posing risks to living organisms and causing not only water pollution but also potentially life-threatening illnesses, such as cancer [5,6,7,8]. Consequently, there is an urgent need for efficient methods to remove or decompose these pollutants, prompting extensive research in this area. Most of these techniques involve incorporating functional materials, such as polymers or hybrid composites, within the performing device [9,10,11,12]. While bulk materials have also been utilized for this purpose, there has been a growing emphasis on focusing on nanoscale functional materials. This is because nanomaterials can provide several benefits ranging from control over the morphology to selectivity and fabrication [13,14,15,16]. Among these nanomaterials, polymers are of particular interest as they offer multiple features (as mentioned above) suitable for similar applications. In polymers, the introduction of a specific chemical functional group (polar or nonpolar) before or post synthesis can help determine the final morphology and selectivity for a particular pollutant and its removal during the water treatment process [17,18,19,20,21,22,23,24]. The chemical and physical properties of a polymer, characterized by a well-designed backbone and desired functional groups, play a considerable role in the fabrication of water purification devices or units [25,26,27]. Functional polymers employed in water treatment processes are modified with polar or ionic groups, such as hydroxyl, carboxylic, amine, phosphonic, and sulfonic groups, among others, to remove a specific pollutant (for example, a metal) [28,29,30,31]. Therefore, when designing functional polymers, the removal pathway in the water treatment process must be considered. To further understand the holistic approach applied to functional polymers concerning their morphology, selectivity, and role in the water treatment processes, it is essential to summarize and highlight all the recent research efforts aimed at developing efficient functional polymers dedicated to pollutant removal. This review provides a detailed presentation of functional polymers, encompassing their distinctive features, microstructures, fabrication techniques, and water treatment properties. This thorough review aims to provide readers with a comprehensive overview of the advancements in functional polymer-based water purification methods to date. Additionally, this review sheds light on prevalent challenges associated with the performance of these polymers, offering a roadmap to address these concerns. The functional polymers under scrutiny are categorized as water-soluble and insoluble, contingent on their repeating units and chemical functional groups. Both types of polymers have found application in various extraction methods, including liquid–liquid extraction, adsorption, and precipitation, based on the distribution of the two phases [32,33]. These methodologies are directly related to the morphology, functionality, and dimensions of the polymeric phase in a membrane [32,33]. Numerous functional polymers have been specifically designed and fabricated for use in membrane preparation. As shown in Figure 1, as a membrane material the functional polymer is an essential component of a water purifier device. Its morphology also plays a decisive role in selective filtration or removal of certain pollutants.

**Figure 2:**
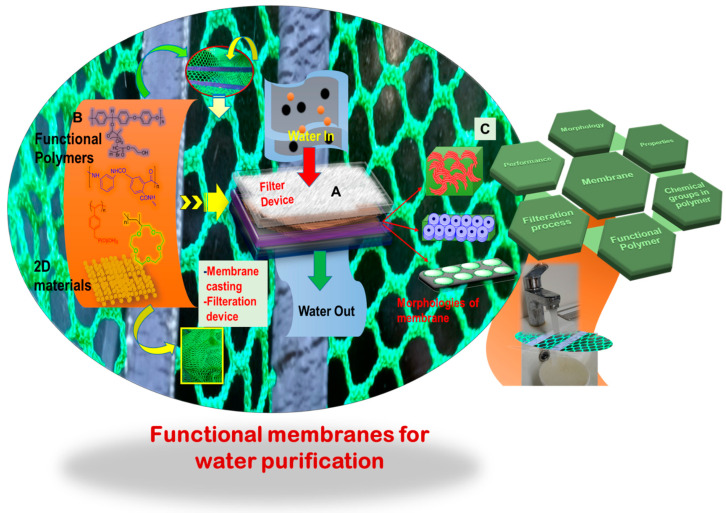
(**A**) A water filter device composed of different components, such as a separating layer, substrate, and a polymeric membrane cast from a relevant functional polymer; (**B**) chemical structures of a few polymers, including their two-dimensional (2D) materials counterparts, highlighting active functional groups crucial for selectivity and filtration, as well as a specific nanostructure suitable for an effective membrane and its processability; and (**C**) various morphologies of polymeric membranes showcasing diverse features, including nanopores, three-dimensional (3D) interconnected channels and homogenous distribution of uniform nanopores, and structure–properties correlation.

A well-controlled and directed morphology further dictates the formation of nanopores within the membrane. The size and shape of these nanopores are crucial for the pathway of water flux. Figure 2 provides a detailed schematic overview showcasing a few polymers, the membrane casting in a device, and the resultant membrane morphology. Common polymers, including polyacrylonitrile (PAN), polyvinylidene fluoride (PVDF), polyethersulfone (PES), polysulfone (PSF), poly(ether ether ketone) (PEEK), poly(amide) (PA), polyurethane (PU), and poly(styrene), along with their derivatives, such as copolymers, have been extensively utilized for membrane filtration [34,35,36,37,38,39,40,41,42,43,44,45,46,47,48]. Crucial considerations when casting or processing membranes in a device involves evaluating the chemical structure, morphology, mechanical, and thermal properties of these polymers [43,44,46,47]. These parameters significantly impact the membrane’s performance in the device. Membranes produced from these synthetic polymers are categorized into the following groups: (1) isotropic or symmetric and (2) anisotropic or asymmetric. This categorization depends on factors like nonporosity, as well as the presence of charged or uncharged polymeric chains (as fabricated through solvent-based or other techniques) [49,50].

**Figure 3:**
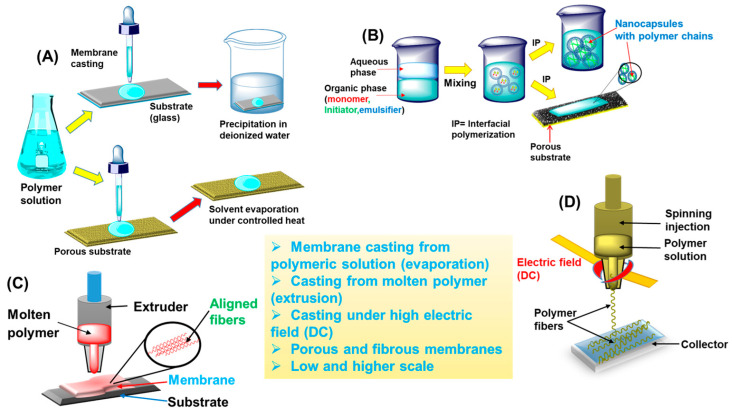
Various techniques for fabricating membranes from polymeric precursors: (**A**) casting a membrane from a polymeric solution on a substrate and evaporating the solvent either via precipitation in a nonsolvent (DI-water) or under controlled heat; (**B**) membrane formation via IP of a monomer at the interface of organic–aqueous phase, resulting in nanocapsules with aligned polymer chains on a porous substrate; (**C**) solvent-free extrusion of a molten polymer, generating aligned fibers in a membrane on a substrate; and (**D**) formation of polymeric fibers and a membrane via electrospinning, achieved by injecting a viscous polymeric solution through needles under electrodynamics or high DC voltage.

In isotropic or symmetric membranes, the porosity and pore size regulate the filtration process, that is, the microfiltration (MF) of molecules or particles with varying sizes or dimensions. Alternatively, nonporous yet dense isotropic membranes have undergone testing for their performance. These membranes allow water to pass through while retaining other “species” or pollutants depending on their solubility and diffusivity. This phenomenon is observed in gas separation, pervaporation, and reverse osmosis (RO) processes. In isotropically charged membranes, such as those used in electrodialysis, the separation process occurs via ionic interactions (regardless of porosity) between the polymeric network’s ionic groups in the membrane and the positively/negatively charged molecules of the impurities [51]. Conversely, anisotropic (asymmetric) membranes exhibit structural or chemical heterogeneity in their networks and are classified into two subgroups: (i) Loeb and Sourirajan membranes and (ii) Thin-film composite (TFC) membranes [52,53,54,55]. Loeb and Sourirajan membranes are fabricated using polymer-forming layers with indistinguishable pore sizes and porosities. In contrast, TFC membranes are prepared employing two different polymers, creating a dense surface layer responsible for separation and a thick, porous layer for providing mechanical support [52,53,54,55]. Several techniques are employed for fabricating polymer-based membranes, serving both research and industrial purposes. Notable methods include interfacial polymerization (IP), sputtering, solution casting, extruding, melt pressing, phase inversion, and electrospinning, all commonly applied for producing polymeric membranes [56,57,58,59,60,61,62]. Figure 3 illustrates a graphical depiction of some of these fabrication techniques. The choice of fabrication process largely depends on the physical and chemical properties of the polymer selected for membrane production. Each of these fabrication techniques possess distinct advantages and disadvantages and may require optimization through factors such as choice of solvent (polar/nonpolar), temperature variation, substrate selection, and the utilization of electrostatic forces. In the phase inversion method, the solid phase of the polymer is converted to either a solution or molten state by adjusting specific parameters, as previously mentioned. For instance, this involves selecting an appropriate solvent and controlling the melting temperature. This technique is further subdivided into immersion precipitation and controlled evaporation precipitation (Figure 3A) [63]. Commercially available membranes are typically fabricated using the immersion precipitation technique. This process involves casting a polymeric solution (containing some additives) onto a suitable substrate, normally glass. Subsequently, the supported polymer is immersed in a nonsolvent agent like pure deionized (DI) water. The immersion-precipitation method is known to yield membranes with higher tensile strength, porosity, and improved hydrophilicity. Additionally, this process results in small-diameter pores that help enhance the thermal shock resistance and volumetric power density of the membrane. In controlled evaporation precipitation, a membrane is cast by depositing a polymeric solution onto a porous substrate and subsequently evaporating the solvent under controlled speed and temperature. By carefully regulating the solvent evaporation from the polymeric solution, a denser, thicker membrane with paper-like characteristics can be obtained. Furthermore, in a slightly modified protocol, when the temperature is significantly increased, a fibrous membrane with an interconnected polymer network can be cast from the solution [64,65]. This process, known as thermally induced phase separation (TIPS), offers numerous advantages, including high porosity, simple processing, easy reproducibility, and a reduced occurrence of defects [65]. In this unique method, IP involves the polymerization of a selected monomer at the interface of water and a suitable solvent, utilizing a porous support as the reaction reservoir. This method leads to the formation of an ultrathin membrane that is highly suitable for ultrafiltration (UF) and RO applications (Figure 3B). Through IP, desirable properties such as high performance, ultrahigh permeability, and a high level of membrane rejection can be introduced during the fabrication process. Additionally, to exclude the use of organic solvents and make fabrication environmentally friendly, the extrusion process has gained widespread use, in which a polymer is subjected to stretching under cold and hot conditions (Figure 3C). In this process, the polymer is heated to its melting point and then extruded through specially designed piston–cylinder equipment, producing polymer aligned fibers when used with a substrate. As these fibers align in a particular direction, the membrane produced with this method displays good mechanical properties, controllable pore size, and thickness.

In the advanced method known as electrospinning, polymeric fibers are produced under an electric field, leading to the evaporation of the solvent and the fabrication of a membrane or film with a considerably large surface area (Figure 3D). In this process, a viscous solution of the polymer is placed in the injector. Furthermore, a strong electric field (DC voltage) is applied across the injector [66]. Once the electrostatic repulsion surpasses the surface tension, the viscous solution flows from the needle as fibers, which are then collected on a designated collector. Through electrospinning, polymeric fibers with multiple arrangements and morphological structures can be created [66]. Table 1 summarizes the aforementioned fabrication techniques, outlining the products formed via each relevant technique, along with their specific properties and applications. This review focuses solely on recent examples of optimized membranes with enhanced performance to elucidate their significant milestones and gain deeper insights. The subsequent section delves into a comprehensive discussion of these membranes, highlighting their distinctive features and examining their perspectives. Earlier, a class of hyperbranched macromolecules known as dendrimers and other polymers have also been explored as membrane materials for similar applications. To describe a few, poly(amidoamine) terminated with sodium carboxylate groups (PAMAM-COONa) was studied for its forward osmosis (FO) to draw solute from the sea water with a relatively high water flux of 9 L m^−2^ h^−1^ [67]. In another report, thermoresponsive magnetic nanoparticles (MNPs) incorporated with a copolymer poly(sodium styrene-4-sulfonate)-*co*-poly(*N*-isopropylacrylamide) (PSSS-PNIPAM) have been shown to draw solute and to extract water from brackish water or seawater via forward osmosis (FO) [68]. Polyacrylic acid and its salt derivatives were also proposed previously for similar applications related to water treatment [69]. In this current review, the major focus is on the most recent state-of-the-art fabrication, developments, and performance of the membrane materials, to provide a comprehensive overview so that a further roadmap can be drawn, if necessary.

## 2. Membranes Based on PA and Its Derivatives

In 1930, at DuPont Wallace, Carothers synthesized a PA, commonly known as nylon [82]. As engineering plastics, PAs are usually synthesized via step-growth polymerization and exhibit high durability and excellent thermal, barrier, and mechanical strengths owing to their rigid backbone. They are used in textiles, the automotive industry, carpets, kitchen utensils, and sportswear.

**Figure 4:**
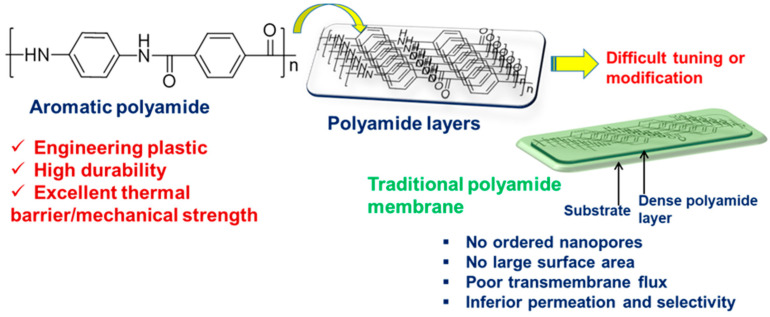
Chemical structure of a representative aromatic PA, categorized as a high-performance or engineering plastic due to its high durability and excellent thermal and mechanical properties. The layers of PA chains in a film are dense and inert to further modifications, necessitating advanced and optimized methodologies for the same.

Figure 4 illustrates the typical structure of an aromatic PA, emphasizing its high-performance attributes and the presence of dense layers in a film or membrane.

Synthetic aromatic PAs are preferred over their aliphatic counterparts because the latter exhibits increased moisture susceptibility, resulting in mechanical weakness and dimensional deformities. Notably, aromatic PAs such as polyphthalamides (PPAs) are processable in the molten state and have accordingly attracted considerable commercial attention. Aromatic PAs have found significant utility exploited as membrane materials owing to their superior hydrolytic and permselective properties. Despite PAs being the gold standard in both academic and industrial efforts for membrane preparation, achieving precise tuning of the PA layer remains a challenging task (Figure 4). For example, the permeability and selectivity of PA-based membranes are directly related to their morphology. To produce the desired morphology wherein PA chains can form dense yet thin layers with a larger surface area (via nanopores), optimization of various parameters becomes crucial. These include the chemical structure of the monomer, fabrication processes, and the structural properties of the porous support. Notably, thin and dense layers are employed to improve permeation within these membranes. Previously, several strategies were introduced for fabricating dense and thin layers of PA chains, including layer-by-layer (LBL) assembly, the addition of interlayers or hydrophilic materials, and 3D printing [83,84]. Studies suggest that aromatic PAs can exhibit porosities ranging from approximately 15  ±  2% to 32  ±  4%. However, increasing the specific surface area via these nanopores enables high-mass crossflow and permeation. For precise nanopore creation, IP using emulsion and template methods is considered highly effective. In IP, a hydrophilic monomer is incorporated and polymerized at the interface, generating a high specific surface area in the resulting polymeric film. Furthermore, the presence of a hydrophilic additive or polymer affects solution viscosity and diffusion rate, directing the formation of a nanoporous morphology in the film [85]. Nevertheless, attempts are ongoing to fabricate defect-free PA membranes, featuring dense layers and ordered nanopores. The pursuit of a perfect membrane aims to establish an optimized water transport pathway, wherein the limits of permeability and selectivity would surpass those of previously reported membranes.

**Figure 5:**
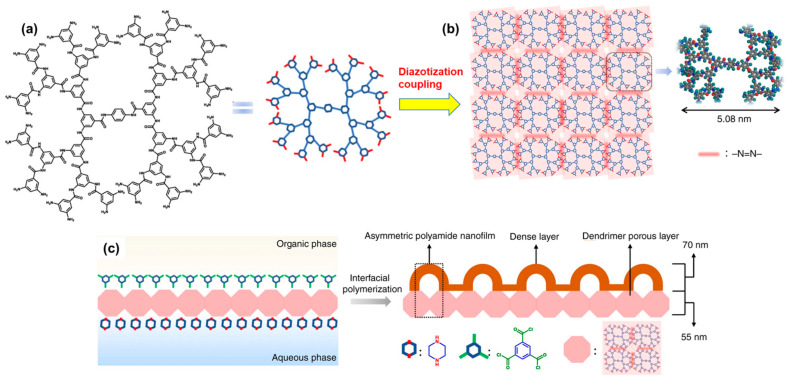
Strategy for creating a double-layered membrane comprising the following: (**a**) hyper-branched–amine-terminated dendrimer which, upon further coupling via diazotization, forms (**b**) a porous layer on a support, followed by (**c**) IP of two different monomers, namely PIP and TMC, conducted on the porous layer. This process aims to produce a dense and thin asymmetric successive layer of PA with an optimized transport pathway and ordered nanovoids. Reprinted with permission from Nature Comm. [86], Copyright 2020 Nature Portfolio. The article is licensed under a Creative Commons Attribution 4.0 International License, which permits use, sharing, adaptation, distribution, and reproduction in any medium or format, as long as you give appropriate credit to the original author(s) and the source, provide a link to the Creative Commons license, and indicate if changes were made.

Recently, a remarkable approach for fabricating asymmetric PA films with well-ordered nanopores and dense layers was reported. This method involves the successive utilization of a PA-based dendrimer and IP of two different monomers to create an efficient double-layered membrane for NF [86]. Figure 5 illustrates a schematic representation of this innovative approach. In the study, PA dendrimers of different generations were employed to create the lower porous layer of a membrane. Since PA dendrimers are hyperbranched and possess a nearly spherical structure, they display peculiar features such as well-defined functional periphery and uniform intramolecular voids or cavities. For this purpose, PA dendrimers with 32-amine terminal groups at the periphery were synthesized and subsequently treated in a salt solution (pH = 1) on the surface of a PSF support. This treatment facilitated their coupling with each other via a diazotization-coupling reaction involving a sodium nitrite solution, culminating in a covalent assembly (Figure 5). Consequently, the surface of the support becomes hydrophilic with minimal defects. In the IP step, these surface-supported covalent assemblies of the PA dendrimer are placed in a solution of piperazine (PIP) for 10 min, followed by immersion in a TMC/n-hexane solution (0.15 *w*/*v*%) for another 30 or 60 s to create a traditional or asymmetric PA dense film. Interestingly, the polar and nanosized cavities of the dendrimers assist the diffusion of PIP molecules, consistently allowing the IP process to smoothly create a uniform film of PA with ordered nanovoids or nanopores [86].

**Figure 6:**
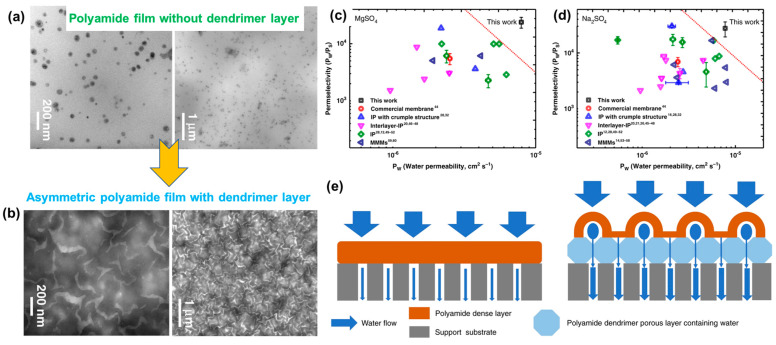
Morphological features of an asymmetric PA membrane: (**a**) TEM micrographs without the dendrimer; (**b**) with the covalent assemblies of dendrimer; (**c**,**d**) permeation and selectivity of an asymmetric membrane produced via IP on dendrimer and other available membranes reported in the literature, including commercial ones; and (**e**) schematic representation illustrating the transportation of water (salt water) across the membrane without and with a dendrimer porous layer, as fabricated via diazotization prior to IP. Reprinted with permission from Nature Comm. [86], Copyright 2020 Nature Portfolio. The article is licensed under a Creative Commons Attribution 4.0 International License, which permits use, sharing, adaptation, distribution, and reproduction in any medium or format, as long as you give appropriate credit to the original author(s) and the source, provide a link to the Creative Commons license, and indicate if changes were made.

Morphological investigations of these double-layered membranes using transmission electron microscopy (TEM) clearly delineated two different layers corresponding to the covalent assembly of dendrimers and asymmetric PAs produced after IP. Figure 6 displays the TEM micrographs showcasing the single-layer PA nanofilms, revealing PA aggregations throughout. However, following the IP process, the structural features of the membrane transformed significantly, characterized by the appearance of uniform nanovoids. At high resolution, an array of interconnected, ordered nanovoids (width of 70–120 nm) was also observed. Further analysis with atomic force microscopy (AFM) and X-ray techniques confirmed the formation of an asymmetric PA membrane, comprising a porous layer of spherical dendrimers and a dense PA layer. Such features are targeted to reduce the transmembrane resistance of water and increase the permeation flux. With regard to the separation performance of these membranes, studies have demonstrated a 3.7- to 4.3-fold higher water flux for various salt solutions (2000 ppm) compared to traditional PA membranes. To further examine the superiority of these double-layered membranes, their permeability and selectivity were tested and compared with the data estimated for different types of PA membranes. These included commercial PA membranes, crumpled PA membranes (IP with crumpled structures), PA membranes prepared using interlayers, traditional IP membranes, and mixed-matrix PA membranes (Figure 6).

The experiments revealed that the permeability (7.9 × 10^−6^ cm^2^ s^−1^) and selectivity (23,877.2) of the membrane for magnesium sulfate or MgSO_4_ exceeded those of the aforementioned membranes. A similarly improved performance (permeability 7.5 × 10^−6^ cm^2^ s^−1^ and selectivity 27,561.3) was also observed for the sodium sulfate or Na_2_SO_4_ solution. As depicted in Figure 6e, the high performance of the membrane can be attributed to the presence of a porous dendrimer layer and a thinner PA layer on the top (Figure 6e). This configuration provides a uniform transformation pathway, facilitating water flux while improving selectivity and salt permeation. In addition, the larger surface area of the nanovoids allows water to permeate efficiently, with increased salt rejection rates (Figure 6e) [86].

In a similar approach, researchers utilized graphitic carbon nitride (g-C_3_N_4_) to construct a double-layered PA membrane through IP, aiming to achieve ultrapermeability and excellent selectivity for water purification [87]. Here, g-C_3_N_4_ functions similarly to the dendrimer mentioned earlier, influencing the IP process by reducing the monomer diffusion at the interface. This reduction results in the formation of a nanoscale–hollow-cone structure within the fabricated membrane.

**Figure 7:**
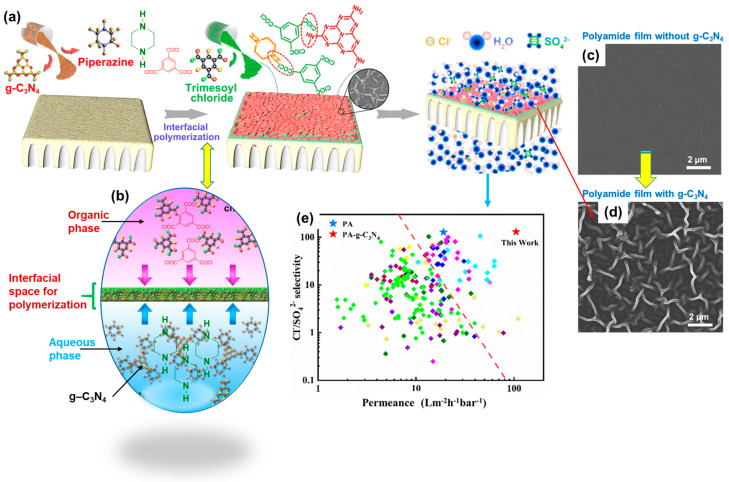
Fabrication process of a membrane utilizing a layer of porous g–C_3_N_4_: (**a**) Deposited on a support in order to conduct IP of two monomers, namely PIP and TMC, leading to polycondensation within the interfacial space. (**b**) Representation of TMC molecules dissolved in the organic phase, with the PIP (remaining in the aqueous phase) adsorbed on the g–C_3_N_4_ surface, resulting in a slow diffusion of PIP molecules through the pores. (**c**) Scanning electron microscopy (SEM) image of a tradition PA film without g–C_3_N_4_ and no porous morphology. (**d**) SEM image of a porous and double-layered PA membrane after IP, displaying a superstructure with ordered nanovoids. (**e**) permeance and selectivity of the membrane. Reprinted with permission from Nature Comm. [87], Copyright 2023 Nature Portfolio. The article is licensed under a Creative Commons Attribution 4.0 International License, which permits use, sharing, adaptation, distribution, and reproduction in any medium or format, as long as you give appropriate credit to the original author(s) and the source, provide a link to the Creative Commons license, and indicate if changes were made.

Figure 7 illustrates a schematic representation of this process. To support PES, a suspension of g-C_3_N_4_ and PIP in an aqueous solution is deposited via dip-coating. After drying, the support coated with g-C_3_N_4_ and PIP is immersed in a solution of TMC in n-hexane, with IP performed by heating the entire assembly to fabricate a porous and ultrapermeable membrane. The surface morphological studies using SEM reveal an ordered structure in the PA membrane post IP (Figure 7c,d). The tilted nanosheets of g-C_3_N_4_ and their interlayer spaces noticeably influence the diffusion of the reacting molecules, resulting in a uniform structure with hollow channels within the membrane (Figure 7d). The ultrapermeability and separation selectivities of the membranes have also been investigated, revealing a superior water permeance of 105 L m^−2^ h^−1^ bar^−1^ and a high selectivity of 130 for Cl^−^ over SO_4_^2−^ (Figure 7e). These outcomes offer promising opportunities for rapid and precise separation of pollutants from water [87]. Further advancements in PA-based membranes include the use of a fluorinated amine monomer (CF_3_(CF_2_)_6_CONH(CH_2_CH_2_NH)_2_CH_2_CH_2_NH_2_) to fabricate membranes via IP with TMC for NF [88]. The perfluoroalkyl groups in the active layer of the PA are responsible for the decreased surface energy. Consequently, the membrane exhibits superior self-cleaning and antifouling properties. These membranes have shown potential suitability for wastewater treatment [89,90].

**Figure 8:**
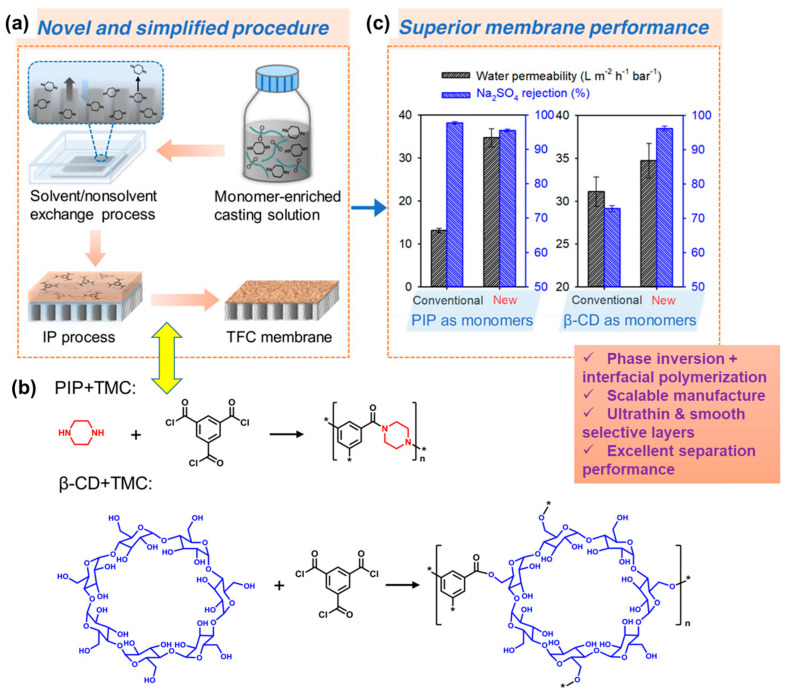
(**a**) Schematic representation illustrating the scalable manufacture of TFC membranes by combining state–of–the–art phase inversion and IP in which (**b**) PIP and β–CD are used as aqueous monomers for conducting IP with a TMC monomer dissolved in the organic phase. (**c**) Performance evaluation of the membrane concerning water permeability and salt rejection. Reprinted with permission from Environ. Sci. Technol. [91], Copyright 2020 American Chemical Society.

In pursuit of a superior membrane based on PAs, a recent report introduced a scalable and versatile fabrication method that combines phase inversion and IP methods for large-scale membrane production [91]. Figure 8 depicts a schematic representation describing this process for fabricating a membrane with ultrathin selective layers. In the first step, a monomer is dissolved in an aqueous phase together with polymer powder (PES) to prepare a homogenous solution that is cast onto a glass substrate. After drying, the glass substrate coated with the monomer and polymer powders is subjected to phase inversion by soaking in a coagulation bath containing water (Figure 8a). A solidified membrane is formed as a result of the solvent/nonsolvent exchange process. A solution of TMC in n-hexane is poured onto the membrane surface to initiate the IP process, resulting in the formation of a TFC membrane (Figure 8a). For comparison, in a separate study, the PIP monomer was replaced with cyclodextrin, which is also soluble in the aqueous phase. The chemical structures of the PIP and cyclodextrin membranes are shown in Figure 8b. The filtration performance tests of these membranes showcase superior water permeability and salt rejection (Figure 8c). The membranes exhibiting excellent performance surpass conventional membranes fabricated exclusively via a single method [91].

PA has been the material of choice for fabricating membranes, particularly when employing aromatic amine monomers like m-phenylenediamine (MPD) for initiating IP on diverse surfaces such as PSF and nylon, by integrating them with a 2D material known as MXene. MXene is known for its large surface area, abundant nanochannels, hydrophilicity, and robust mechanical properties. This led to the development of MXene (Ti_3_C_2_T_x_)–PA membranes fabricated via IP. These membranes exhibit enhanced water flux, antifouling, and chlorine resistance, showcasing benefits for water desalination. These TFC membranes have found application in forward osmosis, water purification, and organic solvent recovery [92,93].

## 3. Membrane Based on Polyvinylidene Fluoride (PVDF) and Its Derivatives

Following the extensive utilization of PAs, PVDF (–(CH_2_CF_2_)_n_–) has emerged as another favored polymer for fabricating membranes due to its impressive mechanical strength, chemical resistance, and thermal stability [94]. Its solubility in various common solvents and easy processability allow the manufacture of flat sheets, hollow fibers, or tubular membranes suitable for MF or UF.

**Figure 9:**
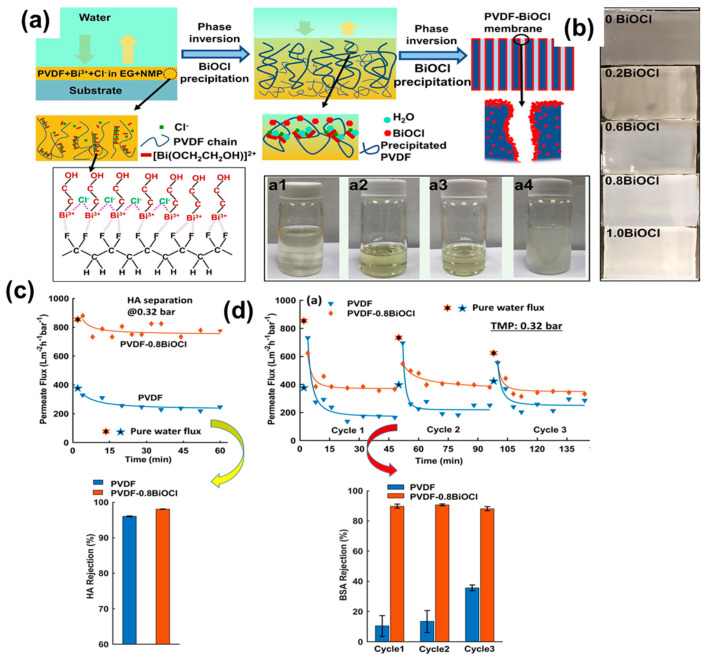
(**a**) Schematic representation illustrating the modified-blending-phase inversion and its mechanism for fabricating PVDF–BiOCl membranes: (a1) Bi^3+^ + Cl^−^ + EG + NMP, (a2) EG + NMP + PVP + PVDF, (a3) Bi^3+^ + EG + NMP + PVP + PVDF, and (a4) Bi^3+^+Cl^−^ + EG + NMP + PVP + PVDF represent various solutions utilized in the studies. (**b**) Images of the membranes with varying BiOCl content formed from the reaction of ethylene glycol, potassium chloride (KCl), and bismuth(III) nitrate (Bi(NO_3_)_3_). (**c**) Changes in permeate flux and rejection ratios of the PVDF and BiOCl-0.8BiOCl membranes using 0.1 g/L humic acid (HA) solutions. (**d**) Permeation flux and rejection for BSA illustrating antifouling properties. Reprinted with permission from Separation and Purification Technology [75], Copyright 2021 Elsevier.

As an advancement, a current modified-phase-inversion fabrication of a PVDF-based membrane is shown in Figure 9. Following this concept, a blend of PVDF, KCl, and Bi(NO_3_)_3_ in EG and NMP is coated on a glass substrate (Figure 9a) [75,95,96]. Before and after the addition of PVDF, the blend appears white and opaque (Figure 9a1–a4). The formation of a complex between Bi^3+^ and EG, and its further interactions with the PVDF backbone may have contributed to this color change. Furthermore, the coated substrate is immersed in DI water to initiate the phase inversion process. This process induces phase separation, resulting in the precipitation of BiOCl and the formation of a superhydrophilic membrane (Figure 9b). Performance tests conducted on these membranes suggest their successful use in water filtration, particularly with substances like HA and bovine serum albumin (BSA), further confirming their roles as separators and antifouling agents (Figure 9c,d, respectively).

**Figure 10:**
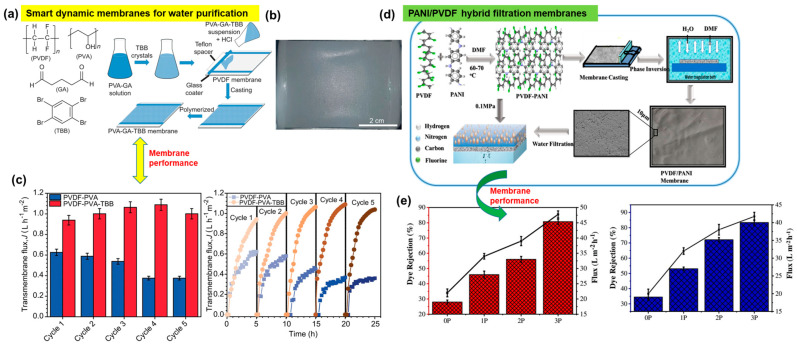
Hybrid and smart membranes based on PVDF. (**a**) PVDF film integrated with a different polymer and dynamic crystalline materials that are capable of sudden expansion or motion under thermal stimulation. (**b**) Microscopic image of the membrane. (**c**) Membrane water flux and durability. (**d**) Hybrid membranes of PANI and PVDF exhibiting (**e**) high dye rejection performance. Reprinted with permission from Nature Commun. [97], Copyright 2023 Nature Portfolio, and Ad. Eng. Mate. [99], Copyright 2022 Wiley-VCH GmbH, respectively. The Nature Communication article is licensed under a Creative Commons Attribution 4.0 International License, which permits use, sharing, adaptation, distribution, and reproduction in any medium or format, as long as you give appropriate credit to the original author(s) and the source, provide a link to the Creative Commons license, and indicate if changes were made.

Further progress towards the development of PVDF membranes has explored more innovative and unique approaches, including the utilization of thermosalient (TS) crystal monomers such as 1,2,4,5-tetrabromobenzene (TBB). This compound is recognized as an energy-efficient–dynamic crystalline material due to its phase transition occurring above room temperature (Figure 10a) [97,98]. These dynamic functional crystals can activate the membrane via mechanical or heat responses, thus increasing the mass transfer. To fabricate these smart, responsive membranes, a layer of PVDF was integrated with a polyvinyl alcohol (PVA) hydrogel (Figure 10a). The fabrication process involves incorporating dynamic crystals of TBB into an aqueous solution containing PVA and glutaraldehyde (GA), resulting in a suspension. Treatment of this suspension with hydrochloric acid or HCl leads to its casting on the porous surface of the PVDF film. Subsequent polymerization between TBB and GA was performed to obtain a smart PVDF–PVA membrane with dispersed TBB crystals (Figure 10a,b). Experiments conducted to evaluate the transmembrane flux of these membranes showcase improved and superior performance compared with the undoped PVDF–PVA membrane (Figure 10c). Even after the fifth operational cycle, the average flux of TBB-doped membranes remains consistently higher, surpassing 160% (Figure 10c). Notably, the phase transition of TBB crystals likely impacts the transport properties upon heating and cooling. Applications of these membranes include distillation and antifouling. Additionally, with regard to hybrid PVDF membranes, another example involves the fabrication of polyaniline (PANI) layers within a PVDF matrix to optimize the hydrophilicity, porosity, antifouling properties, solvent content, and water flux. Figure 10d illustrates a schematic for manufacturing a PVDF–PANI hybrid membrane and its performance characteristics. These membranes have proven effective in the removal of harmful dyes from textile wastewater [99].

In the study, a phase-inversion method is employed to fabricate PVDF membranes with various PANI concentrations (Figure 10d). The addition of PANI (PANI (1–4 wt.%) increases the hydrophilicity of the resulting membrane with reduced pore size, as evidenced by the contact angle and SEM measurements. The operational experimental results demonstrate an enhancement in water flux from 28 to 47 L m^−2^ h, signifying increased hydrophilicity upon addition of PANI to PVDF. Furthermore, the performance tests exhibit high rejection of dye molecules (allura red and methyl orange) (Figure 10e).

**Figure 11:**
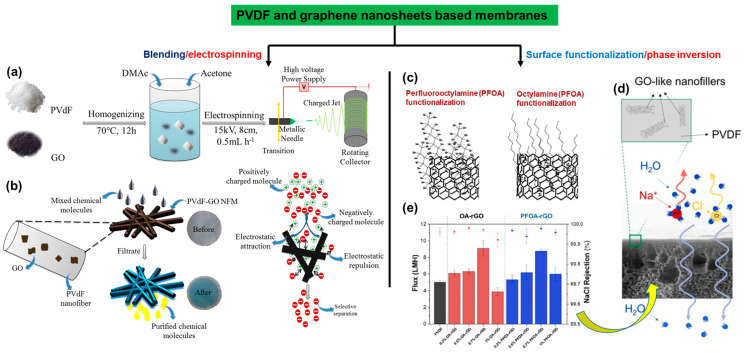
Illustration of two different strategies wherein (**a**) the nanosheets of GO in combination with PVDF were electrospun to generate (**b**) the membranes with knots and with preferential affinity towards positively charged dye molecules. (**c**) In another method, functionalization of graphene nanosheets was implemented to control the degree of hydrophobicity, and subsequently a membrane with (**d**) a PVDF solution is cast via a phase inversion step. (**e**) The performance of the hydrophobic PVDF–GO membrane. Reprinted with permission from Environ. Sci. Technol [102], Copyright 2018 American Chemical Society, and Journal of Environmental Chemical Engineering [103], Copyright 2023 Elsevier, respectively. The article is open access, distributed under the terms of the Creative Commons CC-BY license, which permits unrestricted use, distribution, and reproduction in any medium, provided the original work is properly cited.

Graphene oxide (GO), a widely recognized 2D nanomaterial, has been extensively utilized in various research studies, including its integration with PVDF-based membranes. This integration is due to GO’s advantageous attributes, such as large surface area, chemical inertness, and robust mechanical properties [100,101]. The functional moieties in GO enable surface modification in membranes, essential for simultaneous removing and recycling of industrial organic dyes. To achieve these functional features in a membrane, a simple and straightforward electrospinning approach was employed. This technique facilitated the fabrication of versatile porous structured PVDF–graphene oxide (PVDF–GO) nanofibrous membranes (NFMs) (Figure 11a). The scheme in Figure 11a illustrates the electrospinning of the solution of PVDF and GO that yielded the nanofibers, which were then collected on a rotating collector [102].

In these membranes with porous and uniform geometries, the 3D pores are interconnected through triangular junctions, playing a significant role in the selective separation of various organic dyes (Figure 11b). During filtration, the membranes showcase a selective affinity towards positively charged molecules such that negatively charged organic dye molecules could pass through it and allow selective separation, as is needed for industrial water treatment (Figure 11b). These NF membranes exhibit 99% selectivity for positively charged dyes and 100% rejection of negatively charged dyes from mixed solutions [102].

In a similar study focusing on hydrophobicity and its influence on water flux and salt rejection, GO nanosheets were functionalized covalently and subsequently integrated with PVDF via a phase inversion method to fabricate a hydrophobic membrane with higher efficiency (Figure 11c,d) [103]. Two grafting agents, octylamine (OA) and perfluoroctylamine (PFOA), with distinct degrees of hydrophobicity, were anchored onto the GO surface (Figure 11c). As a result, the increased contact angle and porosity in the membrane directly impact its flux and rejection, reaching 8.8 L m^−2^ h^−1^ and 99.9%, respectively (Figure 11e). These membranes demonstrate promising applications in water [103,104,105,106].

## 4. Membrane Based on Poly (PEEK) and Its Derivatives

**Figure 12:**
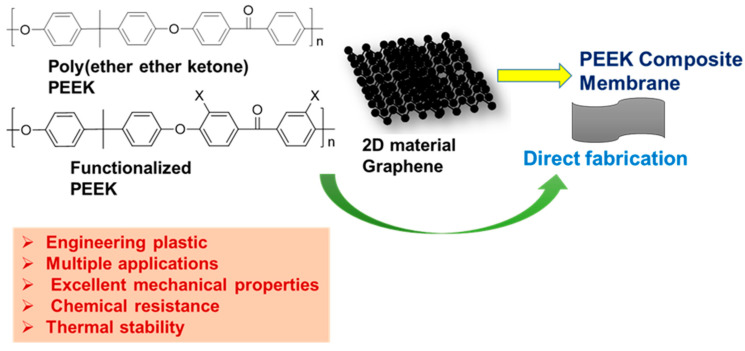
Typical PEEK polymer with its functionalized and composite derivatives that are used for membrane fabrication.

PEEK, a high-performance polymer, exhibits high performance, excellent mechanical properties, chemical resistance, thermal stability, and self-lubrication properties (Figure 12) [107,108,109]. Owing to its processability in various solvents, it has also been used in multiple sectors, spanning aerospace, chemical industry, biomedicine, and membrane technology [108,109,110,111]. By introducing carbon fibers, carbon nanotubes, and various nanoparticles into its matrix, the properties of PEEK can be further tailored, with the resulting composites finding applications in membrane production (Figure 12).

**Figure 13:**
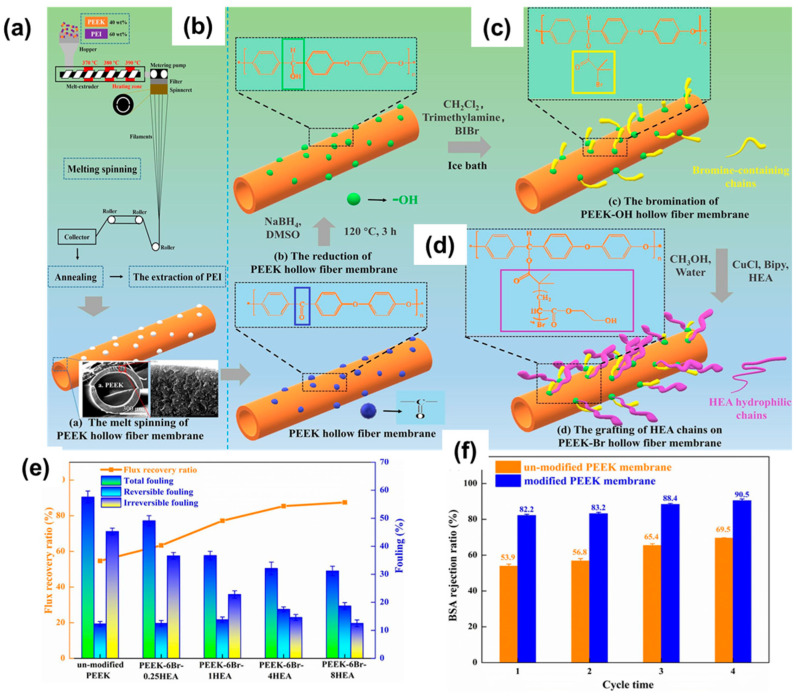
(**a**) Scheme of melt spinning involving a blend of PEEK and PEI to obtain the hollow fibrous membrane (bottom image). This membrane is then subjected to (**b**) reduction to convert the ketone groups into alcohol under sodium borohydride or NaBH_4_, followed by (**c**) esterification on the surface to afford the ATRP active hollow fibrous membrane, which upon further treatment with a hydrophilic monomer, HEA, and a catalyst produces (**d**) brushes (polymeric chains of HEA) on the membrane surface with improved performance, for example, (**e**) improved water flux and (**f**) rejection ratio for BSA. Reprinted with permission from Chemical Engineering Journal [112], Copyright 2023 Elsevier.

In a recent innovative approach, a PEEK hollow fiber membrane (PHFM) was initially fabricated from the blend of PEEK and PEI via melt spinning, as shown in Figure 13a [112]. Next, the ketone groups located on the surface of the hollow fibrous membrane were reduced to alcohol functionalities to anchor an active unit or initiator to create hydrophilic brushes via a controlled polymerization reaction, namely, atom transfer radical polymerization (ATRP) (Figure 13c,d). The analysis revealed that these hollow fibrous membranes were decorated with a hydrophilic layer of 2-hydroxylethyl acrylate (HEA) chains that directly affected the permeability, separation, and antifouling performance of the membranes [112].

Performance tests for these hollow PEEK membranes indicate a high water flux and BSA rejection ratio (Figure 13e,f). The hydrophilic channels resulting from the HEA chains increase the permeability of the membrane. Moreover, the membrane exhibited enhanced antifouling properties attributed to intermolecular repulsion between the fouling agent and the membrane. These modified PEEK base membranes, fabricated via melt spinning followed by covalent functionalization of the surface, are considered promising candidates for wastewater separation and purification.

**Figure 14:**
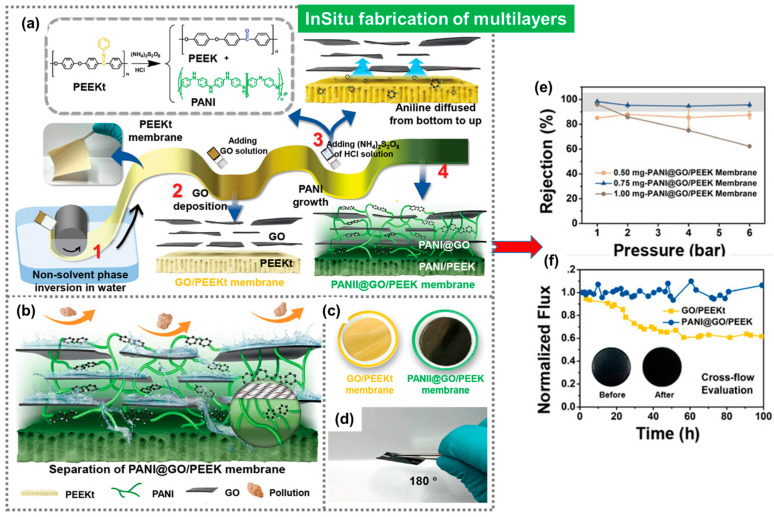
Schematic representation of the fabricating process for the PEEK-based–composite-separating membrane with multilayers made up of (**a**) different chemical components, that is, PANI, PEEKt, and graphene nanosheets integrated in four successive steps; wherein, (1) PEEKt was fabricated via phase inversion, (2) a layer of GO nanosheets was integrated to PEEKt, and (3) layers of PANI were created in situ accompanied with the reformation of PEEKt into PEEK, leading to the (4) PANI@GO/PEEK membrane with multiple interfaces. (**b**) Schematic diagram illustrating the separation process through multiple interfaces. (**c**,**d**) Depiction of the free standing film of the precursor, GO/PEEKt, and the final product, PANI@GO/PEEK, after hydrolysis and oxidative coupling polymerization of PEEKt. (**e**,**f**) The rejection of Rose Bengal sodium (RB) and crossflow of the PANI@GO/PEEK membranes, respectively. Reprinted with permission from the Adv. Sci. [113], Copyright 2023 Wiley-VCH GmbH. The article is open access, distributed under the terms of the Creative Commons CC BY license, which permits unrestricted use, distribution, and reproduction in any medium, provided the original work is properly cited.

In 2023, another novel approach presented a membrane with highly stabilized polymeric interfaces and 2D GO nanosheets, as shown in Figure 14 [113]. Several layers of polyaniline were created via in situ polymerization within the assemblies of PEEK and GO to regulate the pore size of the membrane (Figure 14a). Oxidative polymerization with ammonium persulfate or (NH_4_)_2_S_2_O_8_ led to the anchoring of polyaniline layers. As a result, a modulated microporous structure was developed within the membrane. Membranes with stabilized interfaces and modulated levels of hydrophobicity exhibited flexibility and high separation efficiency (Figure 14b). A rejection of 85% for RB was estimated (Figure 14e). These PANI@GO/PEEK membranes were durable for up to 100 h with a well-maintained water flux (Figure 14f). The performance of these membranes suggested their potential role in large-scale water purification.

## 5. Membranes Based on Porous Organic Polymers and Covalent or Metal Organic Frameworks (COFs/MOFs)

**Figure 15:**
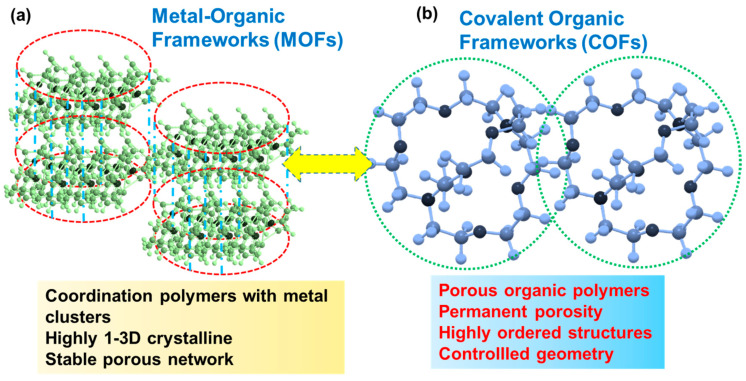
Coordinated and porous organic polymers categorized as (**a**) MOFs and (**b**) COFs, respectively, exhibiting common features such as stable porosity, crystallinity, high order, and controlled geometry.

A new category of hybrid porous materials that combine advantages of both organic polymers and crystalline MOFs have emerged and been utilized for fabricating membranes with greater efficiency for water treatment. These hybrids have garnered attention due to their well-defined supramolecular architectures derived from fundamental building blocks, such as metal clusters and organic ligands. These materials are being explored for the manufacture of membranes designed for water treatment. In these hybrids of polymers and MOFs, a suitable organic ligand is coordinated with a specific metallic center, producing coordinated supramolecular assemblies with long-range order (Figure 15a). These molecular assemblies can be extended into two or three dimensions depending on their linkages [114].

**Figure 16:**
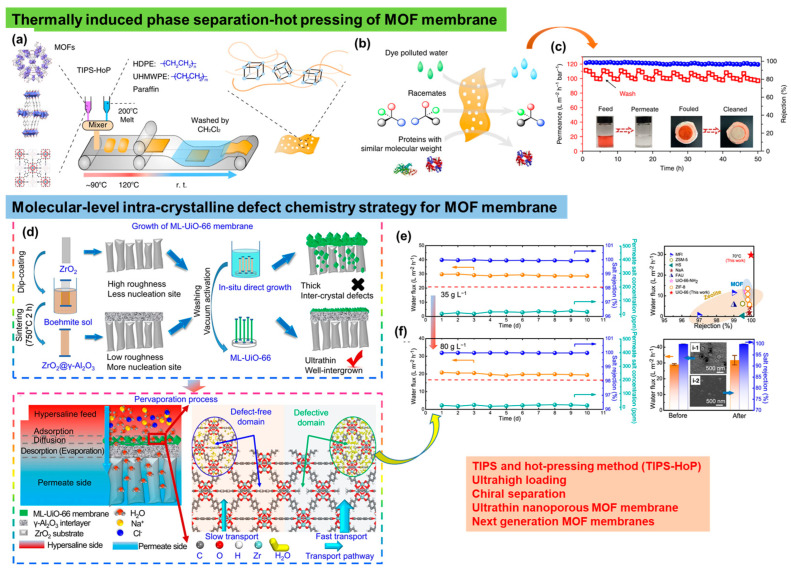
(**a**) TIPS and hot pressing were used to fabricate roll–roll membranes of MOF in which MOF crystals were loaded into the matrix of HDPE (M_w_ > 40,000, melt index = 2.2 g per 10 min) and UHMWPE (M_w_ > 1,500,000) in a molten state which, after cooling to 90 °C, was pressed to manufacture the membranes, with their (**b**) individual selectivity and permeation for different molecules such as dyes, racemates, and proteins in water; (**c**) high performance and recyclability of the membrane with a fouled and cleaned stage; (**d**) ultrathin nanoporous MOF membrane with intra-crystalline defects as created via missing linker within the MOF. (**e**,**f**) The ultrathin membrane with manipulated defects showed a higher water flux and salt rejection as compared to the membrane without any defects. Reprinted with permission from Nature Communi. [116], Copyright 2019 Nature Portfolio, and Nature Communi. [117], Copyright 2023 Nature Portfolio, respectively. These articles are licensed under a Creative Commons Attribution 4.0 International License, which permits use, sharing, adaptation, distribution, and reproduction in any medium or format, as long as you give appropriate credit to the original author(s) and the source, provide a link to the Creative Commons license, and indicate if changes were made.

The key features of MOFs or coordinated macromolecules include ordered open channels, large porosities, predictable pore sizes, and adjustable chemical environments, which offer great opportunities for separation applications. There is a subcategory of polymers that, while lacking metal centers, consist of covalently linked 2D scaffolds with the desired geometries and multiple reactive sites. Correspondingly, it has also attracted considerable attention [115]. These covalently linked 2D scaffolds are known as COFs or porous organic polymers, offering a molecular platform for tailor-made functional materials for membrane development (Figure 15b). Usually, these COFs are crystalline with stable and permanent porosity and are organized in an extended polygon network. These significant features of COFs have allowed researchers to exploit them for membrane manufacturing. Focusing on MOFs, a research group has devised a scalable fabrication method for producing rolls of membranes by using ultra-high-molecular-weight polyethylene, in which MOF particles were interwoven by combining a TIPS and hot pressing (TIPS–HoP) strategy [116]. The combined TIPS–HoP strategy, as shown in Figure 16a, involves several steps: first, blending MOF crystals with high-density polyethylene (HDPE, M_w_ > 40,000), melt index = 2.2 g per 10 min) and ultra-high-molecular-weight polyethylene (UHMWPE, M_w_ > 1,500,000) in paraffin at a higher temperature of 200 °C, allowing the HDPE and UHMWPE to melt; second, placing this molten blend on a moving belt while temperature is brought to ~90 °C for a soft solidification; and third, pressing this soft bulk blend via a roll-to-roll hot pressing at 120 °C resulting in the formation of a membrane which was washed in methylene chloride or CH_2_Cl_2_ to remove the paraffin used in the previous step.

The MOF–PE membranes fabricated via the TIPS–HoP procedure are highly flexible with micron-sized channels, wherein the MOF particles were linked via ultra-high-molecular-weight PE. This renders them as highly efficient separation membranes possessing both high selectivity and high flux (Figure 16b). In the study, several MOFs were used to manufacture roll-to-roll membranes to examine their individual selectivities and permeations for different molecules such as dyes, racemates, and proteins in water (Figure 16b). The permeance tests of these membranes have indicated only 10% decline after 5 h of operation with an outstanding antifouling effect (Figure 16c). The pollutants collected on the membrane surface were eliminated, and the performance of the membrane was further retained (Figure 16c). The high and consistent water fluxes of these membranes with significant rejection make them suitable for water treatment and antifouling sustainable agents [116].

In further advancements related with the MOFs-based membranes, a special strategy to manipulate and rationally design defects was followed to demonstrate how rationally designed defects in a MOF crystal can be used for high-performance membranes (Figure 16d) [117]. In the study, the team used an acetic acid or CH_3_COOH modulator to create intracrystalline defects in the membrane by breaking the linker present in the MOF (UiO-66), which further assisted in producing an ultrathin layer of the modified MOF (Figure 16d, lower panel). As per the performance tests, these ultrathin membranes with well-designed defects showcased an improved water flux, as high as ~29.8 L m^−2^ h^−1^, thereby surpassing the efficiency of other existing membranes for water treatment (Figure 16e,f). Furthermore, the membrane maintained salt rejection (approximately 99.8%) for a longer period of time and can be operated for 12 y at a typical content of salt (Figure 16f). Nevertheless, membranes with rationally designed defects were added to boost the membrane profiles for water purification [117].

**Figure 17:**
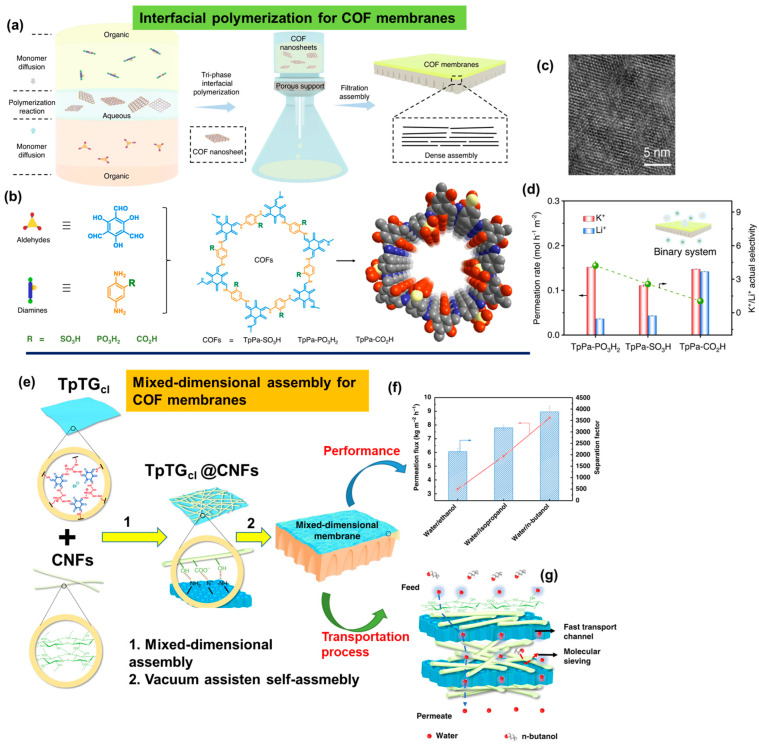
Fabrication of highly porous membranes utilizing (**a**) three–phase (organic–aqueous–organic) IP of (**b**) two different monomers: first, to produce the nanosheets of COFs subjected to a vacuum filtration assembly on a substrate, leading to the (**c**) formation of a dense membrane with a high degree of porous and crystalline networks. (**d**) Water flux ability and selectivity of a COF membrane in a binary mixture. (**e**) Membrane fabricated via the combination and synergism of mixed-dimensional assembly (step 1) and vacuum assisted self-assembly (step 2). (**f**) Permeation properties of the membrane with (**g**) different transport channels in which the feed can run at various speeds or rates. Reprinted with permission from Nature Communi. [119], Copyright 2022 Nature Portfolio, and Nature Communi. [120], Copyright 2019 Nature Portfolio, respectively. These articles are licensed under a Creative Commons Attribution 4.0 International License, which permits use, sharing, adaptation, distribution, and reproduction in any medium or format, as long as you give appropriate credit to the original author(s) and the source, provide a link to the Creative Commons license, and indicate if changes were made.

With the emergence of new functional nanomaterials, further expansion of the above membranes has led to utilization of COFs. They are porous organic polymers which, owing to their robust stability and ordered network of nanochannels, offer superior selectivity and permeation [55,118]. Considering these unique properties, a next-generation membrane was fabricated via IP, wherein an oil–water–oil three-phase system was used as a method for preparing the COF nanosheets. These sheets were subsequently processed as a membrane via the filtration assembly method (Figure 17a) [119]. In the top organic phase, the diamine monomers with three kinds of acid groups, including the 2,5-diaminophenylphosphonic acid group (Pa–PO_3_H_2_), the 2,5-diaminobenzenesulfonic acid group (Pa–SO_3_H), and the 2,5-diaminobenzoic acid group (Pa–CO_2_H) were dissolved. Conversely, in the bottom organic phase, an aldehyde-based monomer (1,3,5-triformylphloroglucinol, Tp) was added, with the middle phase being pure water or an acidic or basic solution (Figure 17a,b).

As the monomers diffused towards the aqueous phase, they underwent polymerization in the interfacial space, resulting in the assembly of COF nanosheets. These nanosheets displayed a dimensional crystalline lattice, as observed with high-resolution TEM (Figure 17c). Subsequently, from the colloidal solution of these nanosheets, a membrane was fabricated via a filtration assembly using a porous support (PTFE with an average pore size of 200 nm) (Figure 17a). For a binary mixture of K^+^/Li^+^, these membranes have shown a high ion permeance with a selectivity of 4.2–4.7. The permeation performance of these membranes was high for single-cation transport, whereas it decreased significantly for binary mixtures (Figure 17d). Nevertheless, the study demonstrated a novel concept of confined cascade separation to successfully enhance the ion transport and separation processes [119].

In a rather simple and straightforward approach, COF membranes based on the 1D cellulose nanofibers (CNFs) and 2D COF nanosheets were fabricated by combining two simple steps: (1) mixed-dimensional assembly of a Schiff base-type COF TpTG_Cl_ (1,3,5-triformylphloroglucinol, Tp and triaminoguanidinium chloride, TG_Cl_) and cellulose nanofibers (CNFs), and (2) vacuum assisted self-assembly of the composites of COF TpTG_Cl_@CNFs on to a PAN substrate (Figure 17e) [120]. The hydroxyl groups present on the surface of the CNFs interacted with the positively charged guanidinium units in the TpTG_Cl_ (COF) framework, leading to dense interlocking in the nanosheets (Figure 17e). Interlocking further increases the mechanical strength and reduces the pore size of the membrane [121]. The separation performance of these membranes can be easily optimized by changing the CNF fraction during fabrication. These membranes have performed with a higher permeation flux of 8.53 kg m^−2^ h^−1^ and a separation factor (α_water/n-butanol_) of about 3876 for the water/n-butanol mixture (Figure 17f). According to the proposed transport mechanism for higher permeation flux, water molecules were preferentially adsorbed onto the membrane surface (Figure 17g). Subsequently, these molecules diffused faster through the well-organized channels of TpTG_Cl_. A successive interlayer of CNF attracted these water molecules at a large concentration with enhanced selectivity via molecular sieving effects. Consequently, a large number of water molecules pass through the next interlayer of TpTG_cl_ at a rapid rate. This entire process leads to a higher water flux, in which the molecules of the respective alcohols are rejected (Figure 17g). Superhydrophilic membranes with interlocked networks and reduced pore sizes are useful for solvent dehydration and the removal of dyes or salts from water [121].

## 6. Conclusions

Drinking water scarcity has become a serious threat to human civilization worldwide. The influx of large-scale pollutants, such as organic dyes, detergents, toxic metals, and even byproducts of fertilizers (after their degradation), as well as next-generation nanoparticles or quantum dots, have adversely affected groundwater and rivers. Polluted water not only increases the risk for the extinction of numerous animal species, but also reduces drinking water availability. To treat polluted water or purify it to eliminate toxic and biohazardous elements, an operational filter device with an integrated membrane is required, which can work at a minimum energy cost (with high efficiency). As a membrane material, a number of well-known polymers such as PAN, PVDF, PES, PSF, PEEK, PA, PU, and poly(styrene) have been fabricated by following various methods and their improvised versions. These methods include IP, sputtering, solution casting, extrusion, melt pressing, phase inversion, and electrospinning. More advanced fabrication processes, such as mixed self-assembly followed by vacuum-induced self-assembly, have also been successfully attempted. With a focus on novel chemistry and controlled properties, such as pore size and interlocked networks, the latest materials, graphene, MOFs, and COFs, have been considered as alternatives to polymers owing to their supramolecular architectures, defects, and robustness. The manipulation of defects within these functional frameworks and the fabrication of membranes have shown promising outcomes. With regard to water flux and rejection of pollutants or salts, membranes based on the above frameworks have displayed superior performance. However, owing to their solid and supercrystalline nature, the processability of these materials remains challenging. Nevertheless, recent progress in polymeric materials and their derivatives for membrane fabrication, as highlighted in this review, has led to a scenario wherein their integration into a water filter device can be accomplished. The quest to conserve or produce drinking water either by purifying it from polluted resources or by desalination of seawater is still ongoing.

## 7. Challenges and Perspectives

Despite the significant advancements in membrane materials summarized above, several issues need to be addressed or minimized for their continued improvement and widespread applications. For example, the fouling and wetting of membranes can impose major problems with regard to their performance and durability. Over time, irreversible fouling can cause the membrane to disintegrate, necessitating a replacement. Membrane wetting is another concern that must be addressed as it can damage the membrane, reducing its lifespan. The sustainability of these membranes is a major focal point due to the negative or harmful effects on the environment. At the industrial scale, the production of membranes must be cost effective; thus, a rather cheaper raw material would serve this purpose. However, the design and development of a cheaper material for membrane fabrication that can work with its maximum water flux and can be deployed under any condition or weather remain major tasks that need to be accomplished. Further challenges may also involve reducing the greenhouse gases and industrial waste generated while producing these membrane materials; thus, more materials based on biomass could be alternatives. Precise control over pore size and functionality, which are directly associated with membrane selectivity, is another domain in which further improvements are required. Although there are a number of commercial membranes sold in the market for water treatment applications, they also suffer from similar problems, for instance, fouling and wetting. As a result, the production cost of more membranes increases significantly. This in turn puts an extra burden on the raw materials as far as their sources are concerned, which inflicts a severe toll on the environment. The energy consumption required to operate the filter device must be reduced. Accordingly, the focus needs to be shifted towards sunlight for supplying power to these filter devices at home or anywhere. Notably, a paradigm shift from polymers to 2D materials for membrane fabrication has already occurred, with some positive outcomes. Although, the materials such as graphene, MOFs, and COFs have fulfilled expectations to a large extent, the production of a commercial membrane based on these materials is still a pipedream. For example, manipulating defects in these materials and placing an appropriate chemical functionality within the pores or channels for selective permeation and rejection are other research-related barriers that need to be overcome. Moreover, these 2D nanomaterials are not easy to process and hence could pose additional challenges when fabricated at the industrial scale.

## Figures and Tables

**Table 1:** Various functional materials with tuned properties used for fabricating their respective membrane by using various techniques and their major applications.

S. No.	Material(s)	Membrane Technique	Parameters	Membrane Characteristics	Applications
1.	Poly(arylene sulfide sulfone) (PASS)	Electrospinning	Conc.=0.27 g·mL^−1^, Voltage = 20 kV, and Speed = 300 rpm	Smallest pore size, highest mechanical property, and best surface wettability	MF, water flux of 747.76 L·m^−2^·h^−1^ and high separation efficiency of 99.9% to 0.2 µm particles [70]
2.	PES	Phase inversion (solvent evaporation)	9 wt.% in dimethylacetamide (DMAc), ZnO-nanoparticles, and Temperature >100 °C	Asymmetry, pores 20–100 nm, and controlled morphology	UF, water flux of over 5600 L m^−2^ h^−1^, and high efficiency [71,72]
3.	PVDF/HEMA	Electrospinning	Voltage = 30 kV and Speed = 150 rpm	Nanofibrous and hydrophilic surface with improved flux	MF (antifouling), water flux 63 L m^−2^ h^−1^, and with a separation efficiency of 98% [73]
4.	PEEK/polyetherimide (PEI)	Extrusion (melt spinning)	40 wt.% PEEK with 60 wt.% PEI, at 360 °C	Hollow fiber membrane, hydrophilic, and recyclable	UF (industrial wastewater and antifouling), with a water flux of 216% [74]
5.	PVDF-bismuth oxychloride (BiOCl)	Phase inversion (blending)	(1:7.5) PVP/PVDF in NMP/EG at RT	Super hydrophilic, Cr (VI) adsorption, and with multifunctional features	UF (antifouling capability), with a water flux of 854 L m^−2^ h^−1^ bar^−1^ [75]
6.	Metal–organic framework (MOF)/ PA	IP	Dispersion of ZIF-L, monomer (EDA/TMC)	TFC (ultrathin)	Pervaporation dehydration [76]
7.	PA (PES support layer)	IP	Fluorinated amine/trimesoyl chloride with SDS as emulsifier	TFC (low surface free energy)	Nanofiltration (NF), wastewater treatment, and with antifouling [77,78]
8.	Polypropylene/PE	Stretching	0.5–3 bar pressure uniaxial/biaxial direction, and melting temperature	Good mechanical properties, thickness controlled, and large scale production	Uranium extraction from sea water [79]
9.	Graphene-PE terephthalate	Track etching	Irradiation/ion beams and acid or alkaline solution	Narrow pore size distribution, high porosity, and low cost	Bioseparation [80]
10.	nano silica-functionalized polydimethylsiloxane (PDMS) ink	3D printing	Homemade 3D printer, micronozzle diameter 150 μm, and curing at 120 °C	High precision, better resolution, low cost, and excellent control over thickness and porosity	Oil–water separation [81]

PA: polyamide; PVDF: polyvinylidene fluoride; UF: ultrafiltration; PVP: polyvinylpyrrolidone; TFC: thin-film composite; IP: interfacial polymerization; EG: ethylene glycol; MF: microfiltration; NMP: 1-methyl-2-pyrrolidinone; RT: room temperature; EDA: ethylenediamine; TMC: trimesoyl chloride; PES: polyethersulfone; SDS: sodium dodecyl sulfate; and PE: polyethylene.

## Data Availability

Data are contained within the article.

## Abbreviations

PASS, Poly(arylene sulfide sulfone); PES, Poly(ether sulfone); PVDF, Poly(vinylidene fluoride); PANI, polyaniline; GO, graphene oxide; ATRP, atom transfer radical polymerization; MOFs, metal–organic frameworks; COFs, covalent organic frameworks; CNFs, cellulose nanofibers; EDA, ethylenediamine; TMC, trimesoyl chloride; SDS, sodium dodecyl sulfate; PPAs, polyphthalamides; LBL, layer-by-layer; IP, interfacial polymerization; NF, nanofilteration; AFM, atomic force microscopy; SEM, scanning electron microscopy; and TEM, transmission electron microscopy.
